# Supplementary material for: Efficient Analysis of Small Molecules via Laser Desorption/Ionization Time-of-Flight Mass Spectrometry (LDI–TOF MS) Using Gold Nanoshells with Nanogaps
Source: Nanomaterials (Basel). 2023 Dec 21;14(1):25. doi: 10.3390/nano14010025 (PMC10781185; doi:10.3390/nano14010025)
Supplement: Supplementary file 1 [file nanomaterials-14-00025-s001.zip › nanomaterials-2706979-supplementary.pdf]

nanomaterials

Supplementary Materials

Efficient Analysis of Small Molecules via Laser Desorption/ Ionization Time-of-Flight Mass Spectrometry (LDI–TOF MS) Using Gold Nanoshells with Nanogaps

Noori Kim [1,†], Yoon-Hee Kim [1,†], Gaon Jo [1], Jin Yoo [1], Seung-min Park [2], Bong-Hyun Jun [1,*] and Woon-Seok Yeo [1,*]

[1] Department of Bioscience and Biotechnology, Konkuk University, Seoul 05029, Republic of Korea; bobbysen@naver.com (N.K.); yoonhees@konkuk.ac.kr (Y.-H.K.); jogaon883@gmail.com (G.J.); jin4261624@gmail.com (J.Y.)
[2] School of Chemistry, Chemical Engineering and Biotechnology, Nanyang Technological University, Singapore 639798, park.seungmin@ntu.edu.sg
[†] These authors contributed equally to this work.
[*] Correspondence: bjun@konkuk.ac.kr (B.-H.J.); wsyeo@konkuk.ac.kr (W.-S.Y.)

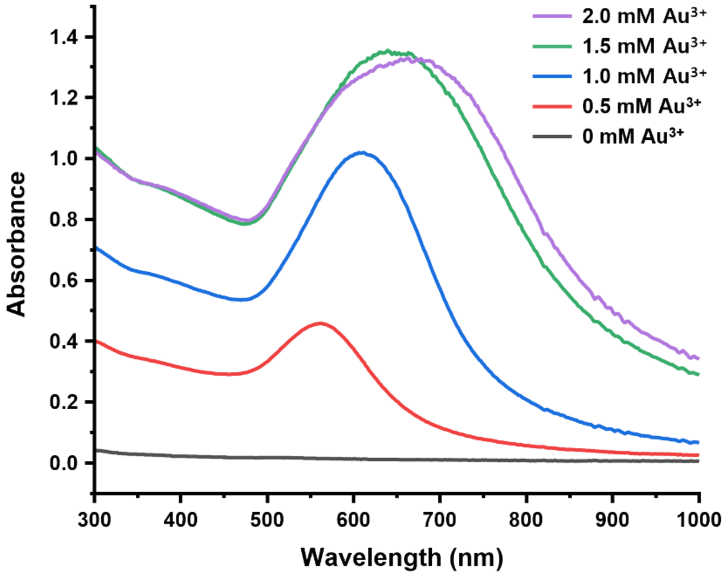

Figure S1. Absorption spectra of SiO_2@Au NGS prepared with different concentration of gold precursor (0, 0.5, 1.0, 1.5, and 2.0 mM Au^{3+}).

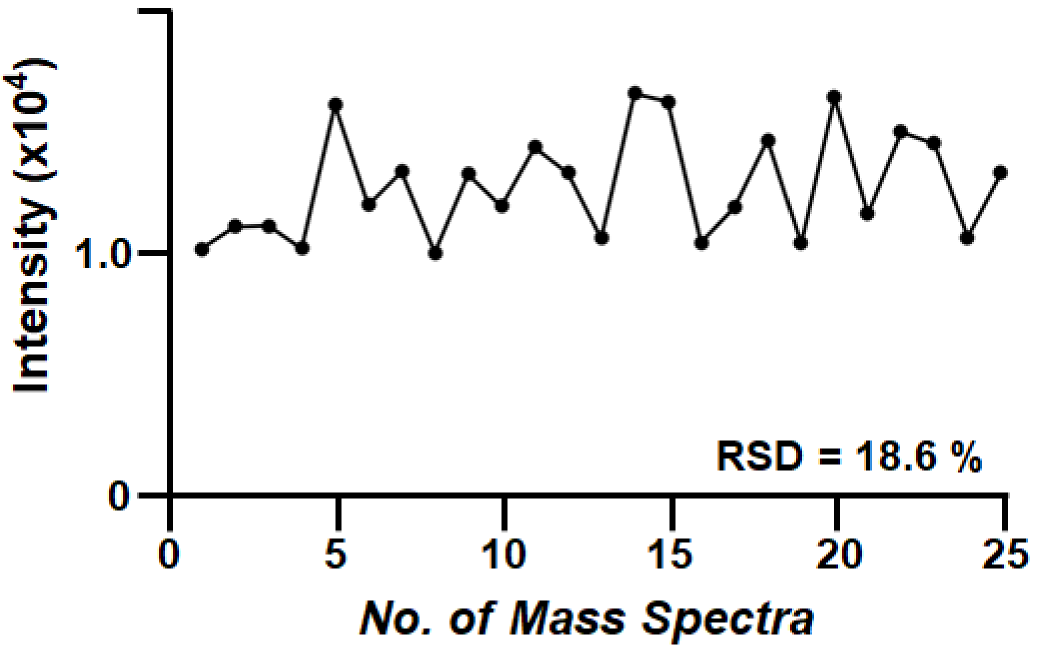

Figure S2. Signal variation of cellobiose. The spectra were obtained from 25 different sample spots using SiO_2@Au $NGS_{2.0}$. The solution of cellobiose (100 μM) was serial diluted with SiO_2@Au $NGS_{2.0}$ to become 100 pmole in 1 μL on the target plate.

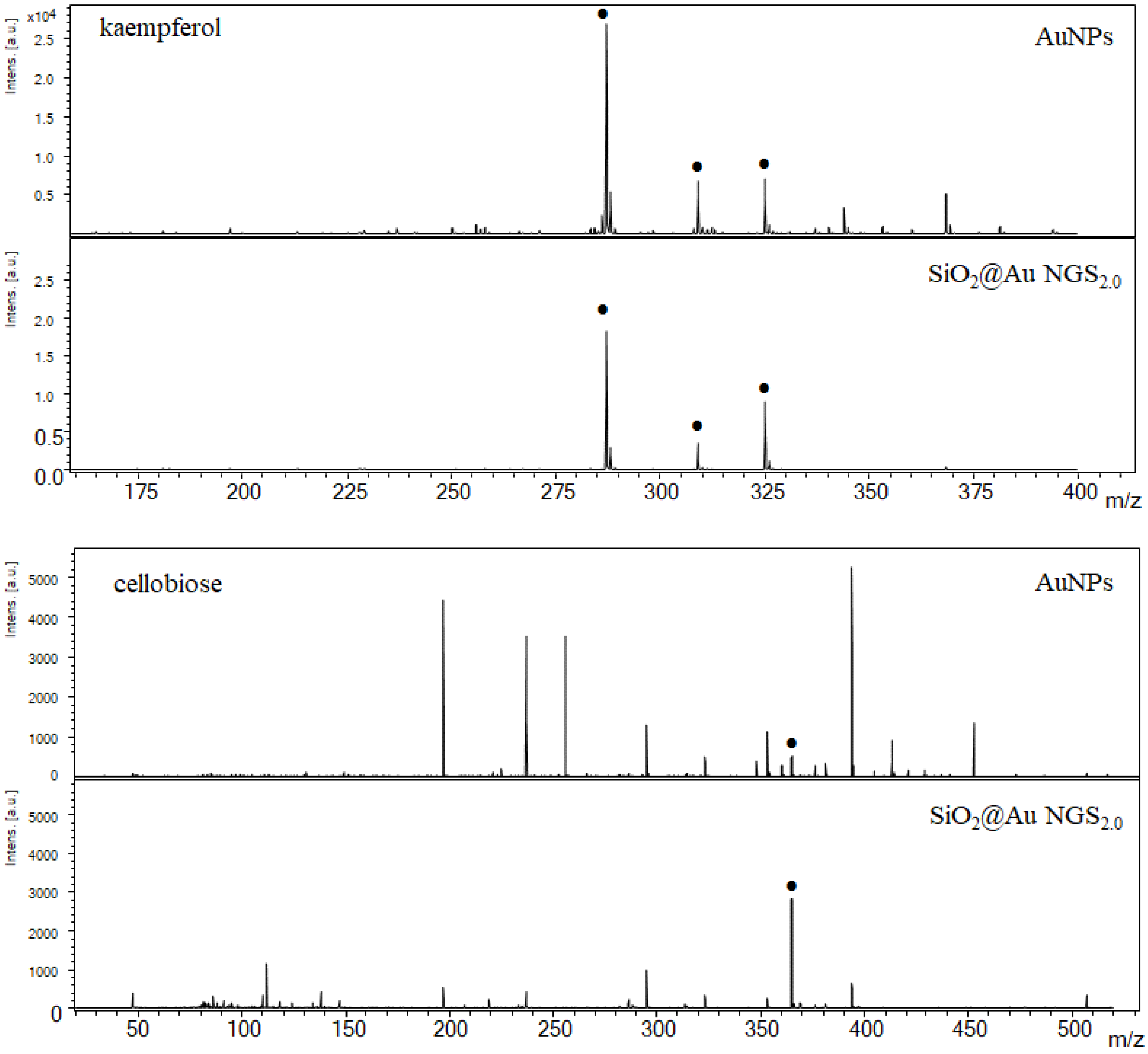

Figure S3. Raw spectra with mass range to ~ 500 for Figure 6a (kaempferol) and Figure 6b (cellobiose). ●: Analyte peak.

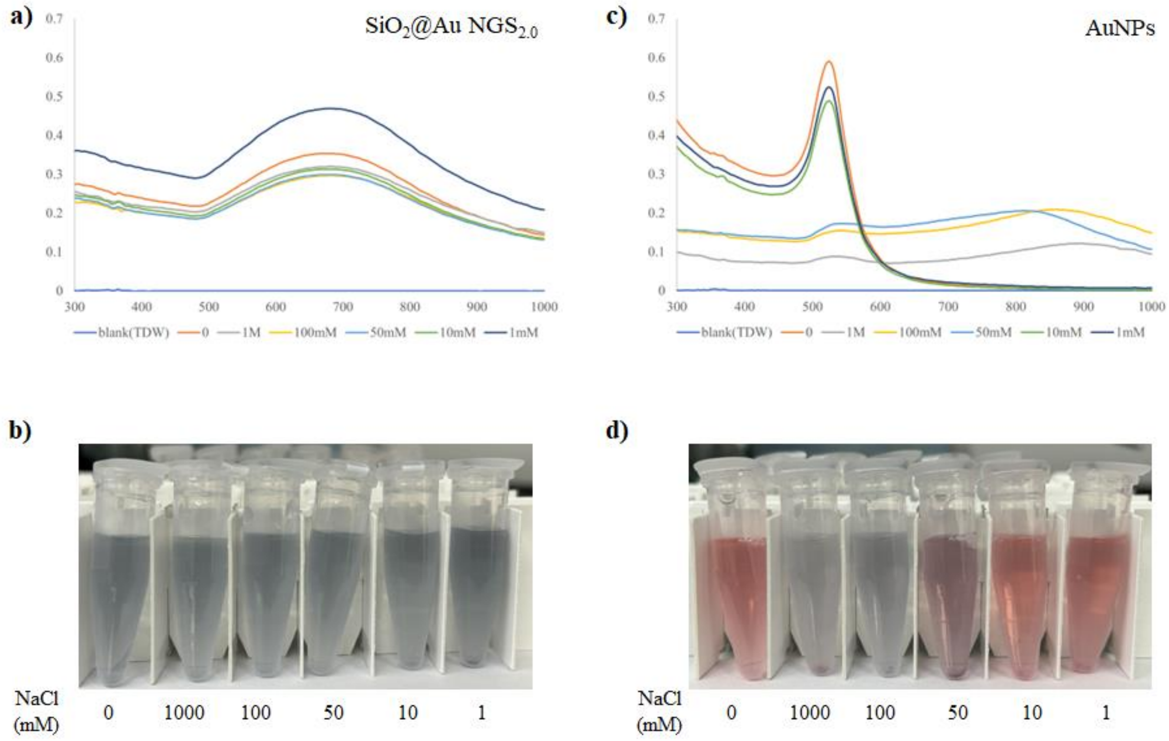

Figure S4. Comparison of colloidal stability in NaCl solutions between SiO_2@Au $NGS_{2.0}$ and AuNPs. Uv/vis spectra (a) and optical photograph (b) of SiO_2@Au $NGS_{2.0}$ showed their excellent colloidal stability, however AuNPs lost their stability at 50 mM or higher salt concentrations (c, d).

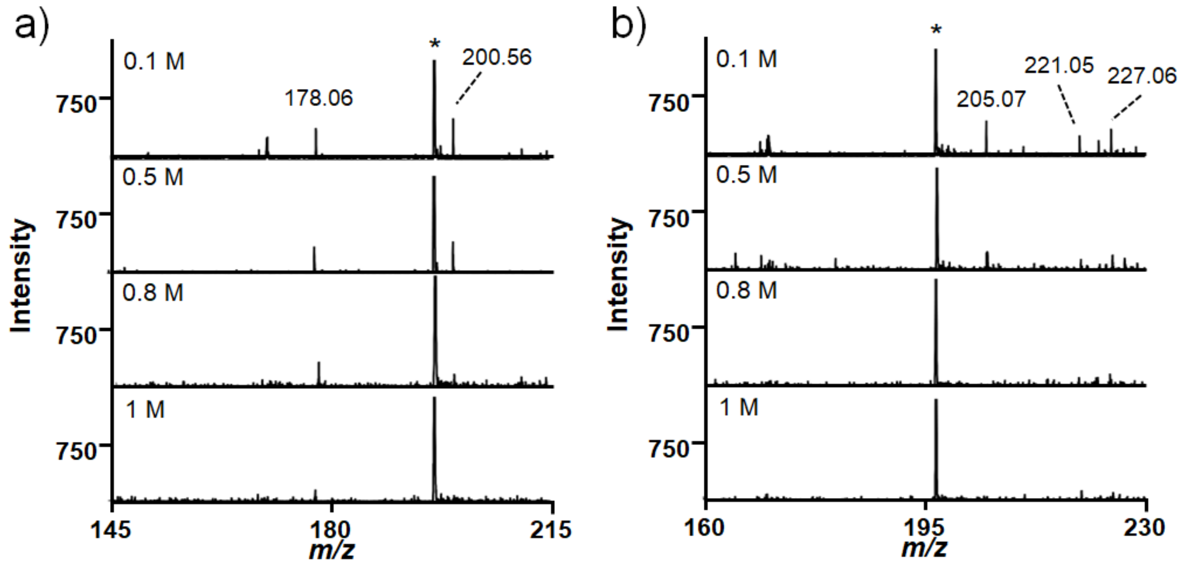

Figure S5. Salt tolerance of the AuNP matrix in NaCl solutions of various concentrations. (a) Histidine (m/z = 178.06, $[M+Na]^+$ and m/z = 200.56, $[M+2Na-H]^+$). (b) Mannitol (m/z = 205.07, $[M+Na]^+$; m/z = 221.05, $[M+K]^+$; and m/z = 227.06, $[M+2Na–H]^+$). *: gold

Table S1. Detailed peak assignments for mass spectra in Fig. 2.

a. Serine

		**0**	**0.5**	**1.0**	**1.5**	**2.0**
$[M+H]^+$	m/z				105.947	105.960
	Intensity				103.99	228.63
	S/N				24.8	52.2
	Resolution				3419	3616
$[M+Na]^+$	m/z		127.953	127.960	127.953	127.970
	Intensity		77.15	135.46	137.66	540.94
	S/N		19.9	33.9	33.8	126.3
	Resolution		5806	5154	3804	4143
$[M+K]^+$	m/z		143.940	143.951	143.941	143.95
	Intensity		31.20	50.62	46.75	151.88
	S/N		8.0	13.7	11.6	37.3
	Resolution		6589	5722	5664	4832

b. Mannitol

		**0**	**0.5**	**1.0**	**1.5**	**2.0**
$[M+H]^+$	m/z					
	Intensity					
	S/N					
	Resolution					
$[M+Na]^+$	m/z			205.138	205.126	205.083
	Intensity			153.56	90.21	2259.55
	S/N			36.3	18.5	385.0
	Resolution			3955	2063	5166
$[M+K]^+$	m/z				221.112	221.056
	Intensity				42.29	1003.41
	S/N				8.8	181.9
	Resolution				2534	4758

c. quercetin

		**0**	**0.5**	**1.0**	**1.5**	**2.0**
$[M+H]^+$	m/z	303.178	303.086	303.097	303.096	303.104
	Intensity	7849.84	4640.69	1378.87	2187.50	2442.22
	S/N	942.2	912.8	219.5	413.0	468.5
	Resolution	1943	3422	4571	3534	3088
$[M+Na]^+$	m/z	325.112	325.050	325.064	325.059	325.064
	Intensity	108.22	277.99	280.27	248.51	245.79
	S/N	12.0	52.1	44.5	47.6	46.7
	Resolution	7324	6742	5079	4402	3897
$[M+K]^+$	m/z	341.118	341.050	341.064	341.061	341.074
	Intensity	408.70	176.34	5157.73	1175.17	1389.15

	S/N	49.2	34.8	850.1	225.4	269.7
	Resolution	5184	5275	4187	4200	3082

d. penta(ethylene glycol)

		**0**	**0.5**	**1.0**	**1.5**	**2.0**
$[M+H]^+$	m/z					
	Intensity					
	S/N					
	Resolution					
$[M+Na]^+$	m/z		261.393	261.303	261.337	261.345
	Intensity		66.88	1613.38	889.41	9112.91
	S/N		18.9	387.7	215.4	2935.6
	Resolution		4935	2935	2460	1913
$[M+K]^+$	m/z		277.386	277.397	277.326	277.327
	Intensity		38.49	889.03	429.51	4166.70
	S/N		11.2	209.9	103.5	2052.1
	Resolution		5468	3870	2778	1881

Table S2. Detailed peak assignments for mass spectra in Fig. 3.

Analyte			$[M+H]^+$	$[M+Na]^+$	$[M+K]^+$
**Aspartic acid**	Aspartic acid	m/z	133.924	155.915	171.895
		Intensity	93.68	893.81	499.65
		S/N	91.8	190.6	110.6
		Resolution	4455	4006	4213
	Glutamic acid	m/z	147.982	169.973	185.955
		Intensity	2064.82	149.77	55.46
		S/N	489.9	37.7	12.7
		Resolution	3759	5969	7375
	Glutamine	m/z	146.975	168.969	184.950
		Intensity	343.0	1776.5	1080.5
		S/N	69.8	330.7	193.1
		Resolution	4380	4278	4181
Oligo Ethylene Glycol	Tri(ethylene glycol)	m/z	151.059	173.201	189.191
		Intensity	129.09	3139.80	873.15
		S/N	29.09	705.2	198.2
		Resolution	4604	3651	4720
	tetra(ethylene glycol)	m/z		217.316	233.303
		Intensity		4850.78	1439.47
		S/N		1105.8	321.8
		Resolution		4878	5992
sugar	Mannitol	m/z		205.083	221.056
		Intensity		2259.55	1003.41
		S/N		385.0	181.9
		Resolution		5166	4758
	Galactose	m/z		203.079	219.211
		Intensity		2580.23	133.95
		S/N		431.4	49.7
		Resolution		4796	6923
Flavonoid	Quercetin	m/z	303.096	325.059	341.061
		Intensity	2187.50	248.51	1175.17
		S/N	413.0	47.6	225.4
		Resolution	3534	4402	4200
	kaempferol	m/z	287.092	309.046	325.083
		Intensity	17905.57	3220.18	7918.13
		S/N	2256.9	382.8	971.0
		Resolution	1706	2858	1930

Table S3. Detailed peak assignments for mass spectra in Fig. 4.

**Mixed analyte**			**[M+H]+**	**[M+Na]+**	**[M+K]+**
**Mixed Amino Acid**	**Glutamine**	m/z		169.044	185.039
		Intensity		76.85	79.78
		S/N		18.3	18.4
		Resolution		3863	5354
	Histidine	m/z	156.054	178.063	194.047
		Intensity	117.43	257.97	140.88
		S/N	28.1	59.2	34.4
		Resolution	3570	3664	4959
	Arginine	m/z	175.116		
		Intensity	416.62		
		S/N	98.6		
		Resolution	3021		
Mixed Oligo Ethylene Glycol	Tri(ethylene glycol)	m/z		173.097	189.082
		Intensity		805.15	176.55
		S/N		118.9	28.6
		Resolution		4200	4313
	Tetra(ethylene glycol)	m/z		217.172	233.154
		Intensity		2641.89	660.77
		S/N		451.5	113.4
		Resolution		4223	5966
	Penta(ethylene glycol)	m/z		261.236	277.211
		Intensity		3291.96	992.08
		S/N		573.4	177.0
		Resolution		4503	6094
Mixed Sugar	Galactose	m/z	180.830	202.982	218.965
		Intensity	81.30	345.46	28.02
		S/N	22.1	95.7	8.0
		Resolution	3067	4651	4731
	Mannitol	m/z		205.007	220.985
		Intensity		805.28	100.91
		S/N		220.7	27.7
		Resolution		4118	4612
	Cellobiose	m/z		365.068	

		Intensity		1289.41	
		S/N		378.7	
		Resolution		4736	
Mixed Small mole-cules	Tryptophan	m/z		226.998	
		Intensity		151.15	
		S/N		39.3	
		Resolution		7742	
	Quercetin	m/z		302.994	340.942
		Intensity		719.32	344.31
		S/N		187.4	82.4
		Resolution		7448	7753
	Cellobiose	m/z		365.049	
		Intensity		2182.82	
		S/N		528.8	
		Resolution		4835	

Table S4. Detailed peak assignments for mass spectra in Fig. 6.

a. Kaempferol

		AuNPs	GO	SiO_2@AuNGS$_{2.0}$
$[M+H]^+$	m/z	287.106	287.147	287.092
	Intensity	28067.01	38384.14	17905.57
	S/N	3670.0	89.2	2256.9
	Resolution	1553	703	1706
$[M+Na]^+$	m/z	309.068	309.123	309.046
	Intensity	6369.35	22250.95	3220.17
	S/N	771.2	59.0	382.8
	Resolution	2290	658	2858
$[M+K]^+$	m/z	325.075	325.443	325.083
	Intensity	6445.01	41243.15	7918.13
	S/N	790.7	140.2	971.0
	Resolution	2512	176	1930

b. Cellobiose

		AuNPs	GO	SiO_2@AuNGS$_{2.0}$
$[M+H]^+$	m/z			
	Intensity			
	S/N			
	Resolution			
$[M+Na]^+$	m/z	365.190		365.177
	Intensity	468.55		2591.05
	S/N	79.8		447.1
	Resolution	5192		6107
$[M+K]^+$	m/z			
	Intensity			
	S/N			
	Resolution			

c. Triethylene glycol

		AuNPs	GO	SiO_2@AuNGS$_{2.0}$
$[M+H]^+$	m/z			
	Intensity			
	S/N			
	Resolution			
$[M+Na]^+$	m/z	172.990	173.039	173.005
	Intensity	10682.83	5769.76	21722.19
	S/N	75.3	14.4	3577.1
	Resolution	690	1042	1005
$[M+K]^+$	m/z	188.983	189.007	188.997

	Intensity	8145.66	7272.58	15184.33
	S/N	51.0	18.7	2431.5
	Resolution	827	890	1124

d. Serine

		AuNPs	GO	SiO_2@AuNGS$_{2.0}$
$[M+H]^+$	m/z			105.953
	Intensity			200.58
	S/N			43.1
	Resolution			4243
$[M+Na]^+$	m/z	127.927		127.959
	Intensity	171.93		322.08
	S/N	28.3		67.0
	Resolution	1416		4592
$[M+K]^+$	m/z	143.910		143.946
	Intensity	92.02		165.96
	S/N	16.1		35.9
	Resolution	2084		4862
